# Development of an opportunistic chest CT-based nomogram for identifying low muscle mass in hospitalized patients with COPD

**DOI:** 10.3389/fmed.2026.1854649

**Published:** 2026-06-17

**Authors:** Hengxing Gao, Xuexue Zou, Yiqing Qu, Qianqian Jiao, Hongbo Li

**Affiliations:** 1 Department of Respiratory and Critical Care Medicine, Binzhou Medical University Hospital, Binzhou, Shandong, China; 2 Medical Integration and Practice Center of Shandong University, Jinan, Shandong, China; 3 Department of Radiology, Binzhou Medical University Hospital, Binzhou, Shandong, China; 4 Department of Pulmonary and Critical Care Medicine, Qilu Hospital of Shandong University, Jinan, Shandong, China

**Keywords:** chronic obstructive pulmonary disease, nomogram, risk prediction, sarcopenia, skeletal muscle density

## Abstract

**Background:**

Low muscle mass is common in patients with chronic obstructive pulmonary disease (COPD) and is associated with adverse clinical outcomes, yet its recognition in routine inpatient care remains limited. We aimed to develop a practical model for identifying hospitalized patients with COPD who were likely to have computed tomography (CT)-defined low muscle mass using routinely available clinical variables and opportunistic chest CT-derived skeletal muscle density (SMD).

**Methods:**

This retrospective single-center study included 265 consecutively hospitalized patients with COPD. Low muscle mass was defined according to sex-specific mean T12 skeletal muscle index (SMI) values derived from the study cohort. Candidate variables were screened using least absolute shrinkage and selection operator (LASSO) regression, and independent factors associated with low muscle mass were identified using multivariable logistic regression. A nomogram was then developed to estimate the probability of low muscle mass. Model performance was assessed by discrimination, calibration, and decision curve analysis (DCA). Internal validation was performed using bootstrap resampling.

**Results:**

The mean age of the cohort was 70.52 years, and 67.6% of patients were male. Five variables were independently associated with low muscle mass: older age, lower body mass index (BMI), higher blood urea nitrogen-to-creatinine ratio (BUN/Cr), lower forced expiratory volume in the first second/forced vital capacity (FEV1/FVC), and lower SMD. The nomogram incorporating these variables showed good discrimination, with an area under the receiver operating characteristic curve (AUC) of 0.823. Calibration analysis showed good agreement between predicted and observed probabilities, and DCA suggested potential net benefit across a clinically relevant range of threshold probabilities.

**Conclusion:**

In this retrospective cohort of hospitalized patients with COPD, a nomogram integrating routine clinical variables and opportunistic chest CT-derived SMD showed promising performance for identifying CT-defined low muscle mass. The model may support early risk stratification and help identify patients who warrant further nutritional, functional, or rehabilitation assessment. External validation is required before clinical implementation.

## 1 Introduction

Low muscle mass and impaired muscle function are highly prevalent in patients with chronic obstructive pulmonary disease (COPD), particularly in older adults and those with advanced disease. These muscle abnormalities are associated with reduced exercise capacity, worse respiratory symptoms, increased hospitalization risk, and poorer long-term survival (1–4). In clinical practice, however, formal muscle assessment is often impractical during acute hospitalization, leaving clinically significant muscle depletion underdiagnosed.

Sarcopenia should not be equated with low muscle mass alone. According to current international consensus definitions, sarcopenia requires the combined assessment of low muscle mass plus low muscle strength and/or low physical performance (5, 6). For the purposes of this study, only computed tomography (CT)-defined low muscle mass was evaluated, and the terms “sarcopenia” and “low muscle mass” are not used interchangeably. Low muscle mass serves as a key surrogate indicator in retrospective and opportunistic screening settings where strength and physical performance cannot be reliably measured.

COPD is a prevalent chronic respiratory disorder characterized by persistent airflow limitation, chronic inflammation, and systemic extrapulmonary effects (7). Among these extrapulmonary manifestations, muscle depletion is increasingly recognized as an important determinant of poor outcomes (8, 9). Patients with COPD frequently exhibit reduced muscle mass and impaired muscle quality, both of which are associated with lower exercise tolerance, more severe symptoms, higher hospitalization rates, and worse long-term prognosis (10, 11). Despite these risks, low muscle mass remains underrecognized in routine inpatient care, largely because comprehensive assessment requires specialized tools or functional testing that are often unavailable during acute hospitalization.

Chest CT is frequently performed in the clinical evaluation of hospitalized patients with COPD. This creates an opportunity for opportunistic body composition assessment without additional radiation exposure or cost (12, 13). Although the L3 vertebral level is traditionally used for muscle quantification, abdominal CT is not routinely obtained in standard COPD care (14). The T12 vertebral level is consistently included in routine chest CT scans and has been clinically validated as a reliable alternative for estimating whole-body muscle status when abdominal imaging is unavailable. Recent studies have confirmed that T12-derived skeletal muscle measurements strongly correlate with whole-body muscle mass and provide independent prognostic value (15–17). In addition to muscle quantity, skeletal muscle density (SMD) derived from CT reflects muscle quality, particularly fatty infiltration, and provides incremental prognostic information (18).

Despite growing recognition of the clinical relevance of muscle abnormalities in COPD, critical knowledge gaps remain. First, most existing studies have focused on prognosis rather than early identification of patients likely to have low muscle mass during hospitalization (19). Second, many diagnostic approaches rely on abdominal CT, dual-energy X-ray absorptiometry, bioelectrical impedance analysis, or formal functional testing, which are not readily available in routine inpatient settings (20). Third, few pragmatic prediction models have integrated routine clinical data, pulmonary function, and opportunistic chest CT-derived muscle quality indicators to identify CT-defined low muscle mass in hospitalized patients with COPD.

Accordingly, the originality and clinical contributions of this study are threefold. First, we developed a prediction model using variables that are commonly available during routine hospitalization, including age, body mass index (BMI), blood urea nitrogen-to-creatinine ratio (BUN/Cr), pulmonary function, and chest CT-derived SMD. Second, we integrated information on muscle quality with routine clinical indicators in a single nomogram to improve bedside risk estimation. Third, the model was based on opportunistic T12 chest CT measurements, which can be obtained from imaging already performed in usual care without additional testing.

The clinical motivation is to provide a simple, actionable screening tool for early risk stratification. This model can help clinicians quickly identify high-risk patients who require formal nutritional, functional, or rehabilitation evaluation-especially in busy clinical settings. Accordingly, this study aimed to develop and internally validate a nomogram for identifying CT-defined low muscle mass in hospitalized patients with COPD.

## 2 Materials and methods

### 2.1 Study design and participants

This was a retrospective, single-center observational study conducted at Binzhou Medical University Hospital between January 2023 and December 2024. The study protocol was approved by the Ethics Committee of Binzhou Medical University Hospital (No. 2024-KY-088) and performed in accordance with the Declaration of Helsinki. Informed consent was waived due to the retrospective and anonymized design.

Consecutive hospitalized patients diagnosed with COPD according to the Global Initiative for Chronic Obstructive Lung Disease (GOLD) criteria were enrolled. All underwent chest CT and pulmonary function testing during hospitalization. Patients were excluded if they had active malignancy, severe hepatic/renal dysfunction, neuromuscular disease, incomplete data, or inability to cooperate with testing.

Given the retrospective and opportunistic design, muscle strength and physical performance were not available for most patients and could not be systematically evaluated. Therefore, the present study used CT-derived low muscle mass as the primary endpoint rather than consensus-defined sarcopenia. Accordingly, the manuscript uses the terms “low muscle mass” or “CT-defined low muscle mass” when referring to the study outcome.

### 2.2 Spirometry assessments

Spirometry was performed in accordance with American Thoracic Society/European Respiratory Society (ATS/ERS) standards (21). FEV1, FVC, and the FEV1/FVC ratio were recorded. The severity of airflow limitation was classified according to the 2024 GOLD recommendations.

### 2.3 Anthropometric and laboratory variables

Age, sex, height, and body weight were recorded at admission, and BMI was calculated. Venous blood samples were collected after overnight fasting. Routine laboratory measurements included BUN, Cr, albumin (Alb), C-reactive protein (CRP), and hemoglobin (Hgb). The BUN/Cr ratio was calculated.

### 2.4 Quantitative radiologic assessment

**Figure 1:**
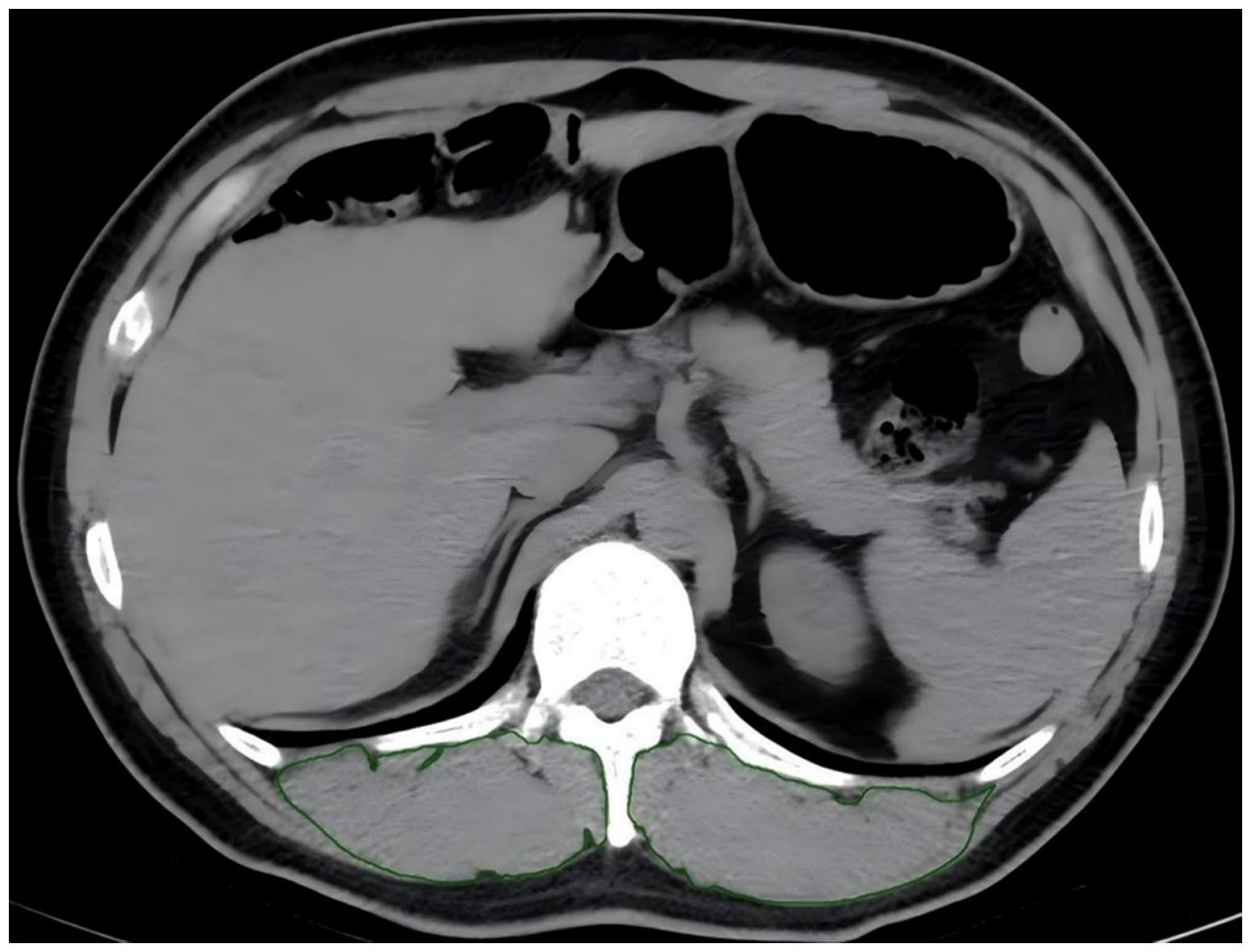
Representative axial CT scan at the T12 level illustrating the semi-automated segmentation of skeletal muscle area (SMA). The regions manually delineated by the green contours denote the paraspinal and intercostal muscle compartments used for quantification.

Chest CT scans were acquired using standard clinical protocols. All measurements were performed at the T12 vertebral level using picture archiving and communication system (PACS) software (22). Skeletal muscle area (SMA) was manually delineated by two independent radiologists who were blinded to clinical data. SMI was calculated as SMA (mm^2^) divided by height squared (m^2^). SMD was defined as the mean attenuation value (HU) within the segmented muscle area (Figure 1).

Notably, both SMI (outcome) and SMD (predictor) were derived from the same T12 CT slice, which may introduce inherent correlation and potential methodological bias. Inter-observer reproducibility was assessed using intraclass correlation coefficient (ICC) in 30 randomly selected cases.

### 2.5 Definition of low muscle mass

Because no universal or externally validated cutoff value for T12 SMI has been established, low muscle mass was defined using sex-specific mean T12 SMI values derived from the present study cohort. This approach has been adopted in some previous opportunistic CT studies, but the resulting thresholds should be considered cohort-specific and exploratory rather than universally applicable diagnostic cutoffs.

### 2.6 Predictor selection and model development

Candidate variables included age, BMI, BUN/Cr ratio, FEV1/FVC, SMD, and other clinically relevant factors. Least absolute shrinkage and selection operator (LASSO) regression was used for variable selection. Multivariable logistic regression was performed to identify independent predictors associated with low muscle mass. A nomogram was constructed based on the final model.

### 2.7 Model performance and validation

Model discrimination was evaluated using the area under the receiver operating characteristic curve (AUC). Calibration was assessed using calibration curves and the Hosmer-Lemeshow test. Clinical utility was evaluated using decision curve analysis (DCA). Internal validation was performed using bootstrap resampling (1,000 repetitions), whereas external validation was not performed owing to the single-center design.

### 2.8 Statistical analysis

Continuous variables were expressed as mean ± standard deviation (SD) or median (interquartile range, IQR). Categorical variables were expressed as numbers and percentages. Between-group comparisons were performed using *t* test, Mann–Whitney U test, chi-square test, or Fisher’s exact test, as appropriate. A two-sided *p* value <0.05 was considered statistically significant. All analyses were performed using SPSS 25.0 and R 4.4.1.

## 3 Results

### 3.1 Demographic and clinical characteristics

**Figure 2:**
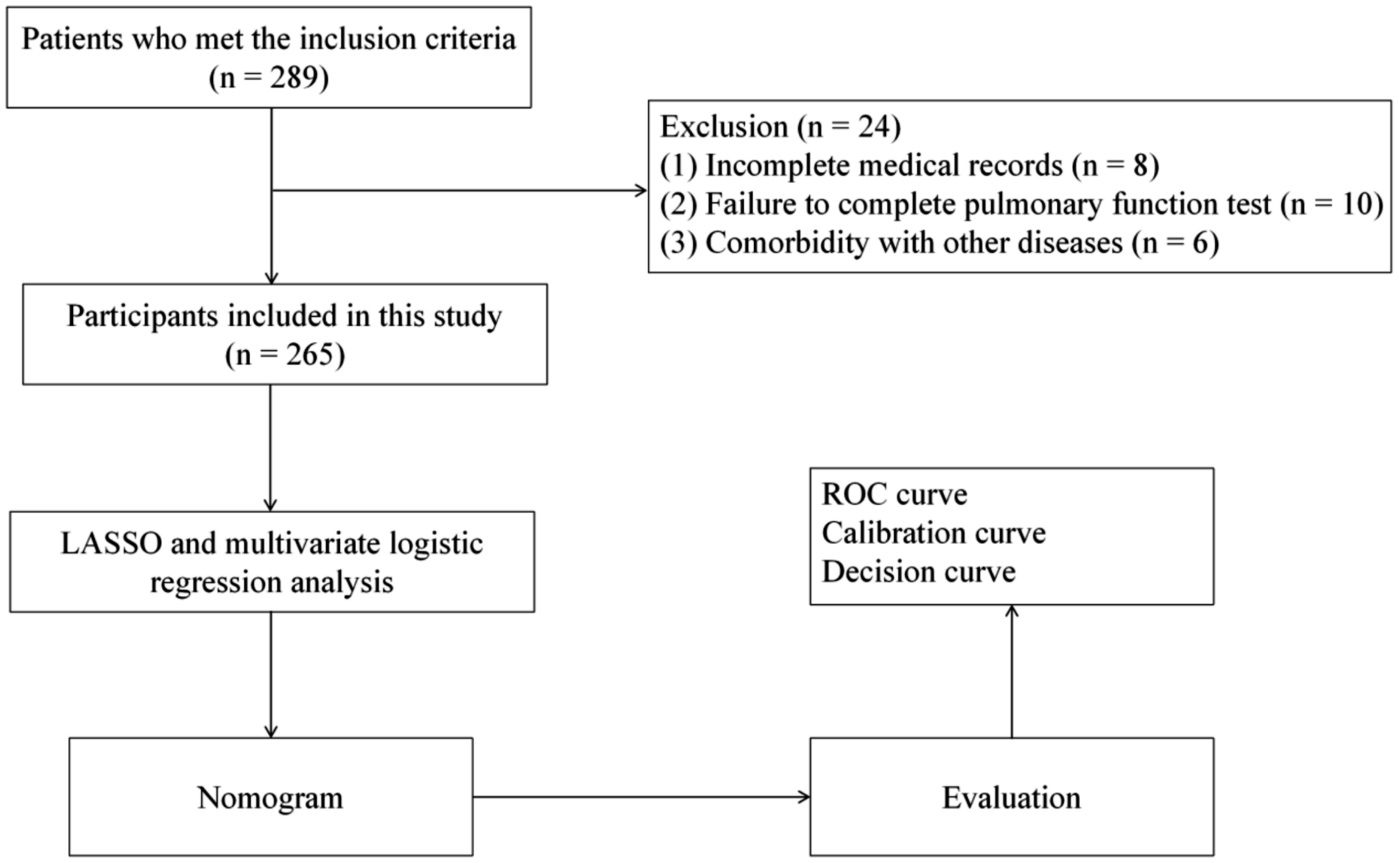
Flowchart depicting the systematic screening, inclusion, and exclusion process of the study population.

A total of 289 patients were screened for eligibility. After excluding 8 patients with incomplete medical records, 10 who were unable to complete spirometry, and 6 with comorbid conditions likely to substantially affect muscle mass, 265 patients were included in the final analysis (Figure 2). The mean age of the cohort was 70.52 ± 7.71 years, and 179 patients (67.6%) were male. The mean T12 SMI was 1117.83 ± 254.32. Overall baseline demographic, laboratory, radiologic, and pulmonary function characteristics are summarized in Table 1.

**Table 1:** Baseline clinicodemographic and clinical characteristics of the analytical cohort (*n* = 265).

Characteristics	Value or no. of patients
Age, years	70.52 ± 7.71
Sex, *n* (%)	
Female	86 (32.4%)
Male	179 (67.6%)
BMI, kg/m^2^	
Female	22.32 ± 4.69
Male	22.54 ± 4.00
SMI	1117.83 ± 254.32
SMD, HU	41.90 (33.30–47.90)
WBC, ×10^9^/L	7.30 (5.75–9.75)
Neutrophils, ×10^9^/L	5.50 (4.00–7.85)
Lymphocytes, ×10^9^/L	1.20 (0.80–1.60)
Hgb, g/L	136.60 ± 16.78
NLR	5.25 (2.90–8.05)
BUN, mmol/L	5.44 (4.19–6.65)
Cr, μmol/L	59.80 (49.65–71.00)
BUN/Cr	93.23 ± 30.68
Alb, g/L	37.85 ± 5.47
CRP, mg/L	10.10 (3.35–46.60)
FEV1, ml	0.85 (0.65–1.16)
FEV1% pred	37.70 (27.90–53.45)
FVC, ml	1.92 ± 0.67
FVC% pred	63.97 ± 18.69
FEV1/FVC	49.91 ± 10.62
PaO2, mmHg	76.14 ± 20.76
PaCO2, mmHg	45.06 ± 8.30
SaO2%	95.00 (93.00–96.50)

Values are presented as mean ± SD, median (IQR), or *n* (%).

BMI, body mass index; SMI, skeletal muscle index; SMD, skeletal muscle density; WBC, white blood cells; Hgb, hemoglobin; NLR, neutrophil to lymphocyte ratio; BUN, blood urea nitrogen; Cr, serum creatinine; Alb, serum albumin; CRP, C-reactive protein; FEV1, forced expiratory volume in the first second; FEV1% pred, percentage of predicted FEV1; FVC, forced vital capacity; PaO2, arterial partial pressure of oxygen; PaCO2, arterial partial pressure of carbon dioxide; SaO2%, arterial oxygen saturation.

### 3.2 Comparison between patients with normal and low SMI

Because no universally accepted reference threshold for T12 SMI is currently available, patients were categorized into a normal SMI group (*n* = 120, 45.2%) and a low SMI group (*n* = 145, 54.8%) using sex-specific mean SMI values derived from the study cohort. Compared with the normal SMI group, patients in the low SMI group were older (72.06 vs. 68.67 years, *p* < 0.001), more often female (37.9% vs. 25.8%, *p* = 0.036), and had a lower BMI (20.90 vs. 24.37 kg/m^2^, *p* < 0.001).

With respect to CT-derived muscle characteristics, the low SMI group demonstrated significantly reduced SMD (39.80 vs. 43.47 HU, *p* = 0.003). Laboratory findings revealed that participants in the low SMI cohort had diminished Hgb (*p* = 0.022), serum Cr (*p* = 0.013), and Alb levels (*p* = 0.002), as well as a higher BUN/Cr ratio (*p* = 0.003) and CRP levels (*p* = 0.012). In addition, pulmonary function was poorer in the low SMI group, as reflected by significantly lower FEV1 (*p* < 0.001), percentage of predicted FEV1 (FEV1% pred) (*p* = 0.002), and FEV1/FVC values (*p* < 0.001). No significant between-group differences were observed for leukocyte counts, neutrophil to lymphocyte ratio (NLR), BUN levels, or arterial blood gas parameters (all *p* > 0.05) (Table 2).

**Table 2:** Intergroup comparison of baseline parameters and laboratory findings stratified by SMI status.

Characteristics	Normal SMI (*n* = 120)	Low SMI (*n* = 145)	*p*-value
Age, years	68.67 ± 8.60	72.06 ± 6.53	< 0.001
Sex, *n* (%)			0.036
Female	31 (25.8%)	55 (37.9%)	
Male	89 (74.2%)	90 (62.1%)	
BMI, kg/m^2^	24.37 ± 4.03	20.90 ± 3.73	<0.001
SMD, HU	43.47 (36.62–48.85)	39.80 (30.05–47.15)	0.003
WBC, ×10^9^/L	7.30 (6.02–9.37)	7.40 (5.40–9.90)	0.499
Neutrophils, ×10^9^/L	5.25 (4.20–7.73)	5.70 (3.85–7.95)	0.984
Lymphocytes, ×10^9^/L	1.20 (0.80–1.70)	1.10 (0.80–1.55)	0.416
Hgb, g/L	139.19 ± 16.38	134.46 ± 16.85	0.022
NLR	5.14 (2.80–8.61)	5.36 (3.03–7.64)	0.847
BUN, mmol/L	5.36 (4.11–6.42)	5.53 (4.24–6.78)	0.382
Cr, μmol/L	62.95 (52.33–76.05)	58.00 (48.60–67.95)	0.013
BUN/Cr	87.15 ± 27.92	98.26 ± 32.01	0.003
Alb, g/L	38.97 ± 5.91	36.93 ± 4.92	0.002
CRP, mg/L	6.85 (3.23–33.55)	15.20 (3.94–56.25)	0.012
FEV1, ml	0.92 (0.73–1.35)	0.79 (0.63–1.02)	< 0.001
FEV1% pred	43.10 (32.38–56.57)	33.80 (26.70–47.90)	0.002
FVC, mL	1.99 ± 0.67	1.85 ± 0.66	0.072
FVC% pred	65.53 ± 18.30	62.69 ± 18.96	0.220
FEV1/FVC	52.44 ± 10.63	47.82 ± 10.17	< 0.001
PaO2, mmHg	75.26 ± 17.15	76.87 ± 23.36	0.517
PaCO2, mmHg	44.48 ± 7.47	45.54 ± 8.93	0.305
SaO2%	95.00 (93.00–96.75)	95.00 (93.00–96.50)	0.782

Values are presented as mean ± SD, median (IQR), or *n* (%).

BMI, body mass index; SMI, skeletal muscle index; SMD, skeletal muscle density; WBC, white blood cells; Hgb, hemoglobin; NLR, neutrophil to lymphocyte ratio; BUN, blood urea nitrogen; Cr, serum creatinine; Alb, serum albumin; CRP, C-reactive protein; FEV1, forced expiratory volume in the first second; FEV1% pred, percentage of predicted FEV1; FVC, forced vital capacity; PaO2, arterial partial pressure of oxygen; PaCO2, arterial partial pressure of carbon dioxide; SaO2%, arterial oxygen saturation.

### 3.3 Predictor selection and multivariable analysis

**Figure 3:**
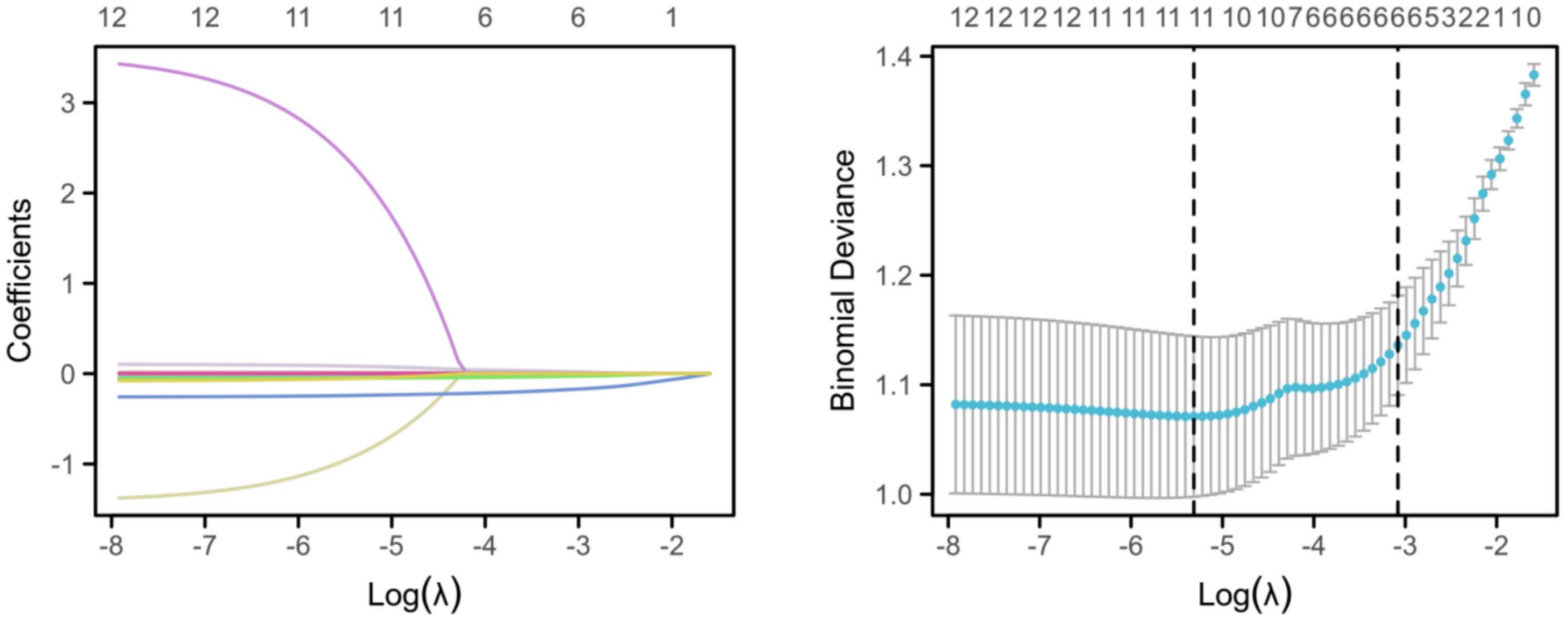
Feature selection leveraging the Least Absolute Shrinkage and Selection Operator (LASSO) algorithm. (Left) Plot of LASSO coefficients against the log(lambda) sequence. (Right) Identification of the optimal tuning parameter (lambda) via 10-fold cross-validation to mitigate overfitting.

Following the implementation of the LASSO regression algorithm to mitigate dimensionality risks, six variables with non-zero coefficients were identified (Figure 3). Collinearity diagnostics revealed variance inflation factors (VIF) ranging from 1.030 to 1.210, confirming the absence of significant multicollinearity (Table 3). Multivariable logistic regression delineated that advanced age (odds ratio, OR = 1.059, *p* = 0.007) and an elevated BUN/Cr ratio (OR = 1.011, *p* = 0.031) were independent factors associated with CT-defined low muscle mass. In contrast, BMI (OR = 0.779, *p* < 0.001), FEV1/FVC (OR = 0.970, *p* = 0.044), and SMD (OR = 0.949, *p* < 0.001) served as independent protective factors. Notably, serum Alb did not maintain statistical significance in the multivariable framework (*p* = 0.217; Table 4).

**Table 3:** Multivariable linear regression analysis of the associations between clinical parameters and SMI.

Variables	*β*	*t*	95% CI	*p*-value	VIF
Age	−0.203	−3.816	−10.157 to −3.242	< 0.001	1.166
BMI	0.443	8.247	20.260–32.970	< 0.001	1.185
Alb	0.075	1.476	−1.161 to 8.103	0.141	1.054
BUN/Cr	−0.131	−2.617	−1.903 to −0.269	0.009	1.030
FEV1/FVC	0.088	1.676	−0.370 to 4.596	0.095	1.140
SMD	0.260	4.791	3.246–7.777	< 0.001	1.210

BMI, body mass index; SMI, skeletal muscle index; CI, confidence interval; SMD, skeletal muscle density; BUN, blood urea nitrogen; Cr, serum creatinine; Alb, serum albumin; FEV1, forced expiratory volume in the first second; FVC, forced vital capacity.

**Table 4:** Multivariable logistic regression analysis identifying independent predictors of low muscle mass in patients with COPD.

Variables	B	OR	95% CI	*p*-value
Constant	5.668	289.320		0.013
Age	0.058	1.059	1.015–1.105	0.007
BMI	−0.250	0.779	0.716–0.847	< 0.001
Alb	−0.034	0.966	0.915–1.020	0.217
BUN/Cr	0.011	1.011	1.001–1.022	0.031
FEV1/FVC	−0.031	0.970	0.941–0.999	0.044
SMD	−0.052	0.949	0.923–0.977	< 0.001

BMI, body mass index; CI, confidence interval; SMD, skeletal muscle density; BUN, blood urea nitrogen; Cr, serum creatinine; Alb, serum albumin; FEV1, forced expiratory volume in the first second; FVC, forced vital capacity.

### 3.4 Correlation analysis

**Figure 4:**
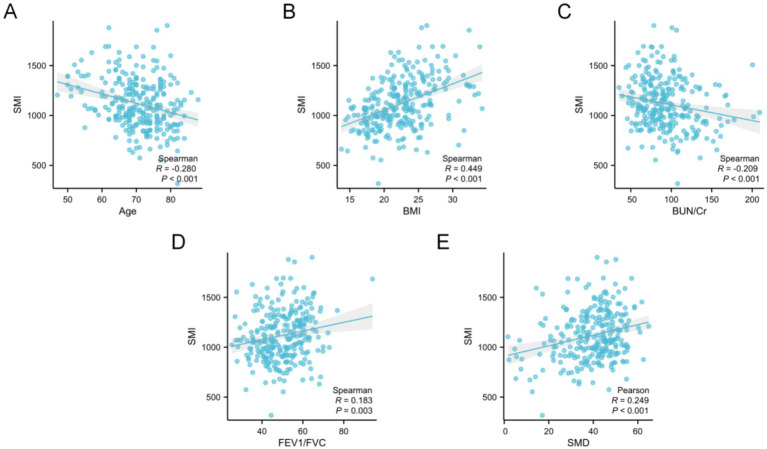
Bivariate correlation analyses between skeletal muscle index (SMI) and independent clinical determinants. **(A)** Age; **(B)** BMI; **(C)** BUN/Cr ratio; **(D)** FEV1/FVC; and **(E)** SMD. Spearman/Pearson coefficients and *p*-values are provided for each correlation. BMI, body mass index; SMD, skeletal muscle density; BUN, blood urea nitrogen; Cr, serum creatinine; FEV1, forced expiratory volume in the first second; FVC, forced vital capacity.

T12 SMI exhibited a significant negative correlation with age (*r* = −0.280, *p* < 0.001) and the BUN/Cr ratio (*r* = −0.209, *p* < 0.001). Positive correlations were observed between SMI and BMI (*r* = 0.449), FEV1/FVC (*r* = 0.183), and SMD (*r* = 0.249) (all *p* < 0.005), as visualized in Figure 4.

### 3.5 Nomogram performance

**Figure 5:**
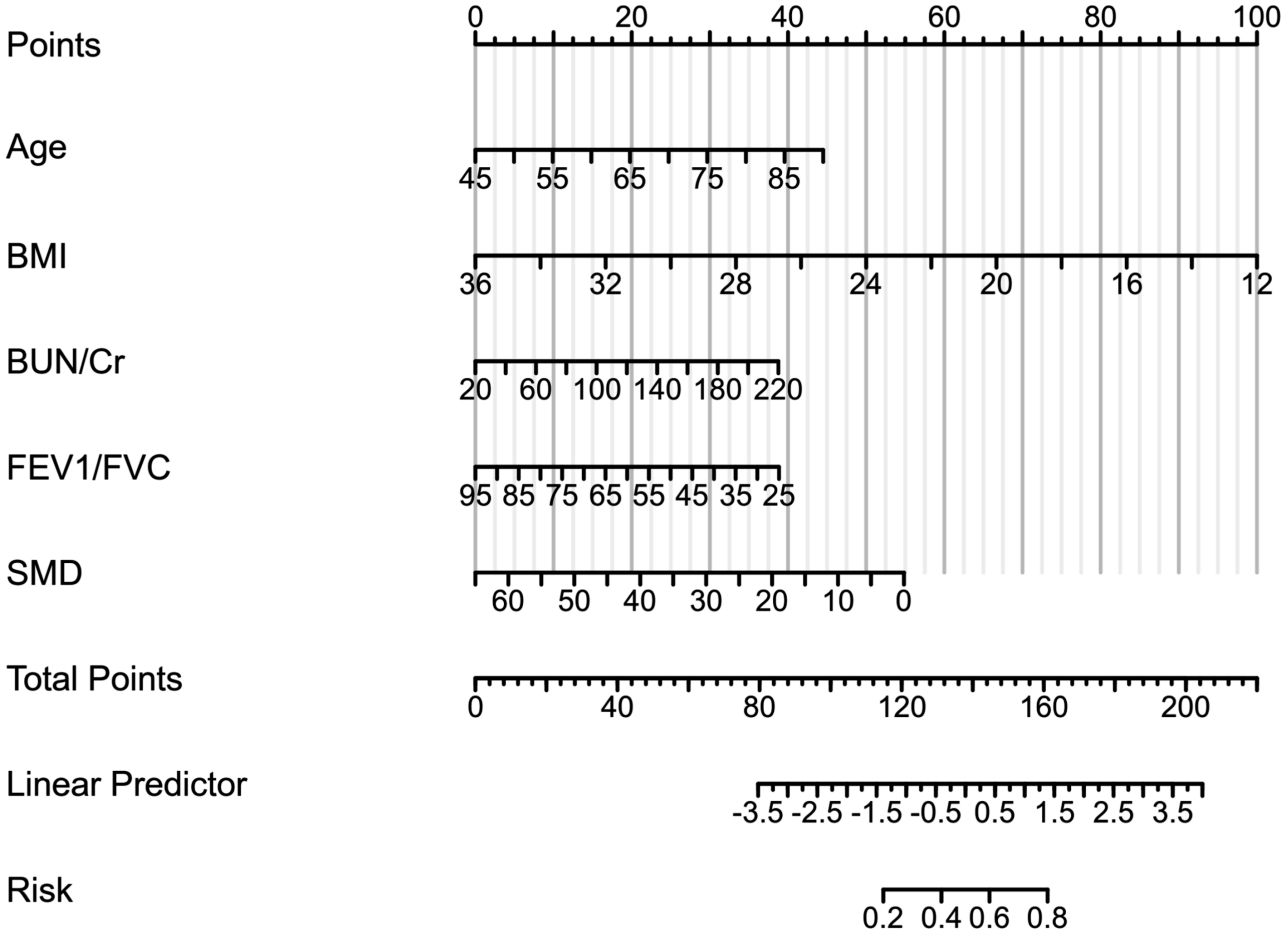
An integrative clinical nomogram engineered for the personalized risk stratification of low muscle mass in patients with COPD. This predictive tool synergizes five multidimensional parameters, including demographics (Age), anthropometrics (BMI), laboratory biomarkers (BUN/Cr ratio), pulmonary function (FEV1/FVC), and opportunistic radiologic metrics (SMD). To utilize the nomogram, clinicians can determine the point value for each variable, sum them to obtain a “Total Points” score, and map this score to the “Risk” axis to estimate an individual patient’s probability of low muscle mass. BMI, body mass index; SMD, skeletal muscle density; BUN, blood urea nitrogen; Cr, serum creatinine; FEV1, forced expiratory volume in the first second; FVC, forced vital capacity.

**Figure 6:**
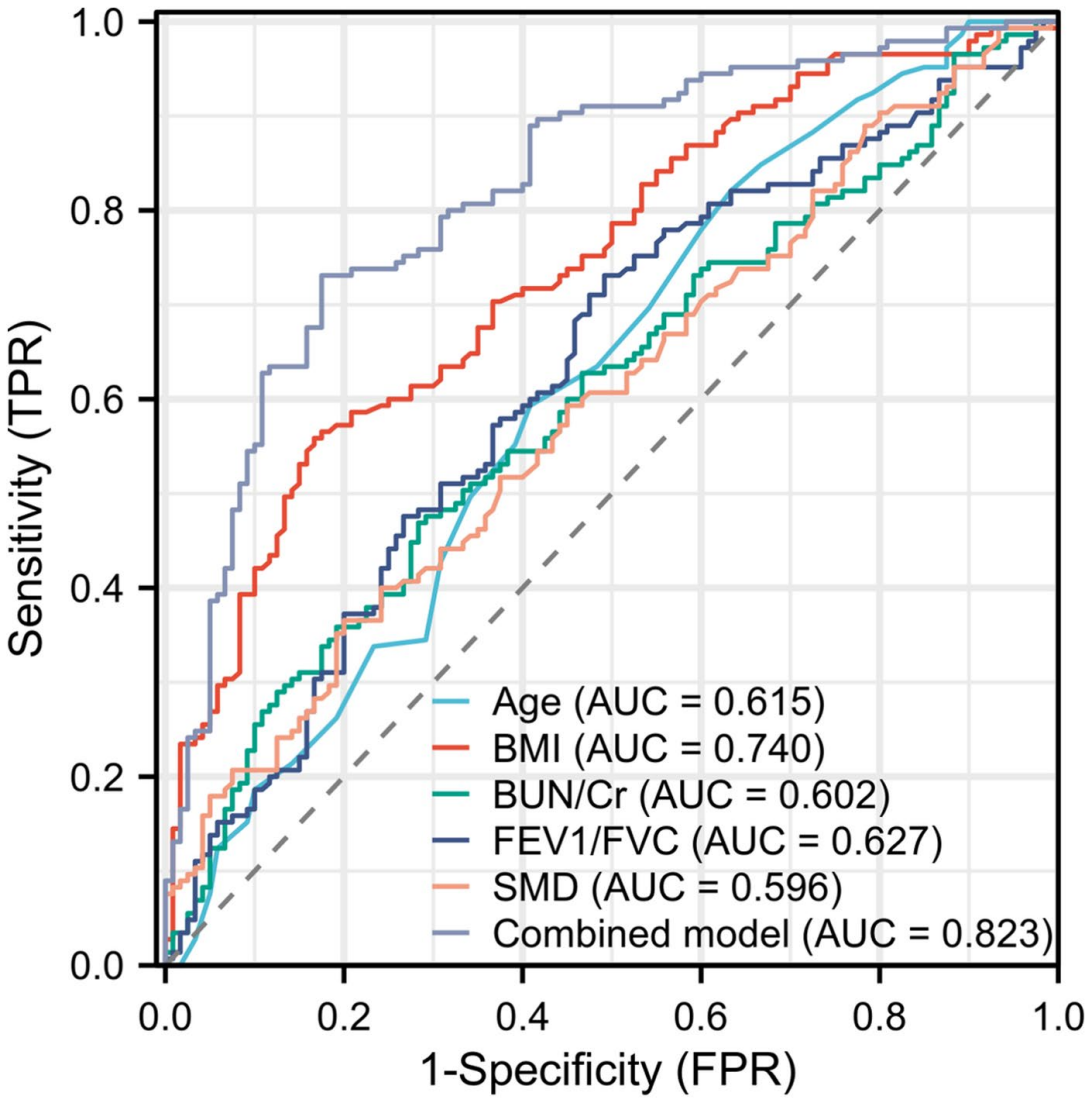
Receiver operating characteristic (ROC) curves evaluating the discriminatory performance of the integrative nomogram compared to single biomarkers. BMI, body mass index; SMD, skeletal muscle density; BUN, blood urea nitrogen; Cr, serum creatinine; FEV1, forced expiratory volume in the first second; FVC, forced vital capacity.

**Figure 7:**
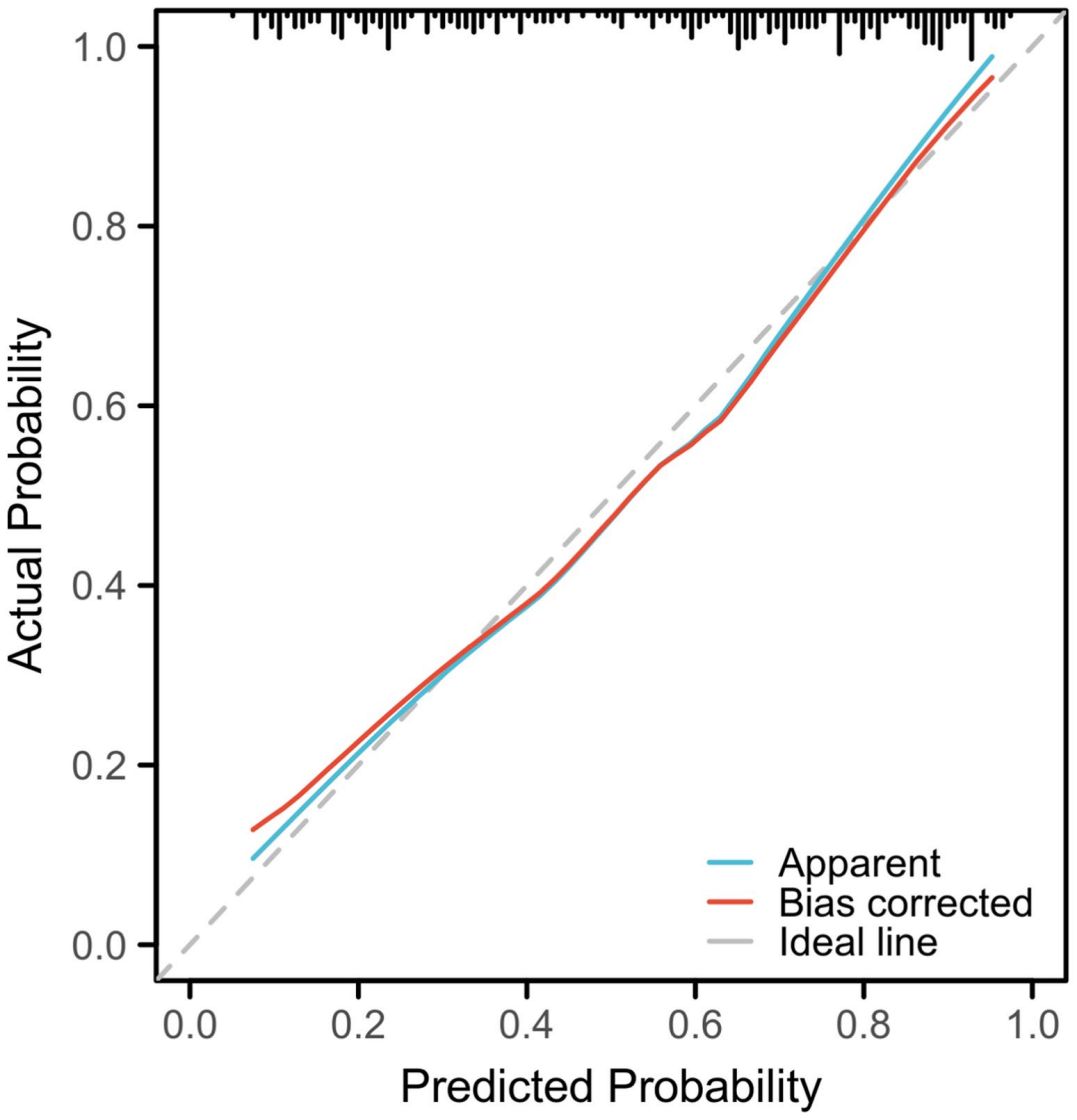
Calibration curve assessing the predictive fidelity of the nomogram. The diagonal dashed line represents the ideal prediction, while the solid line indicates the actual performance of the model.

**Figure 8:**
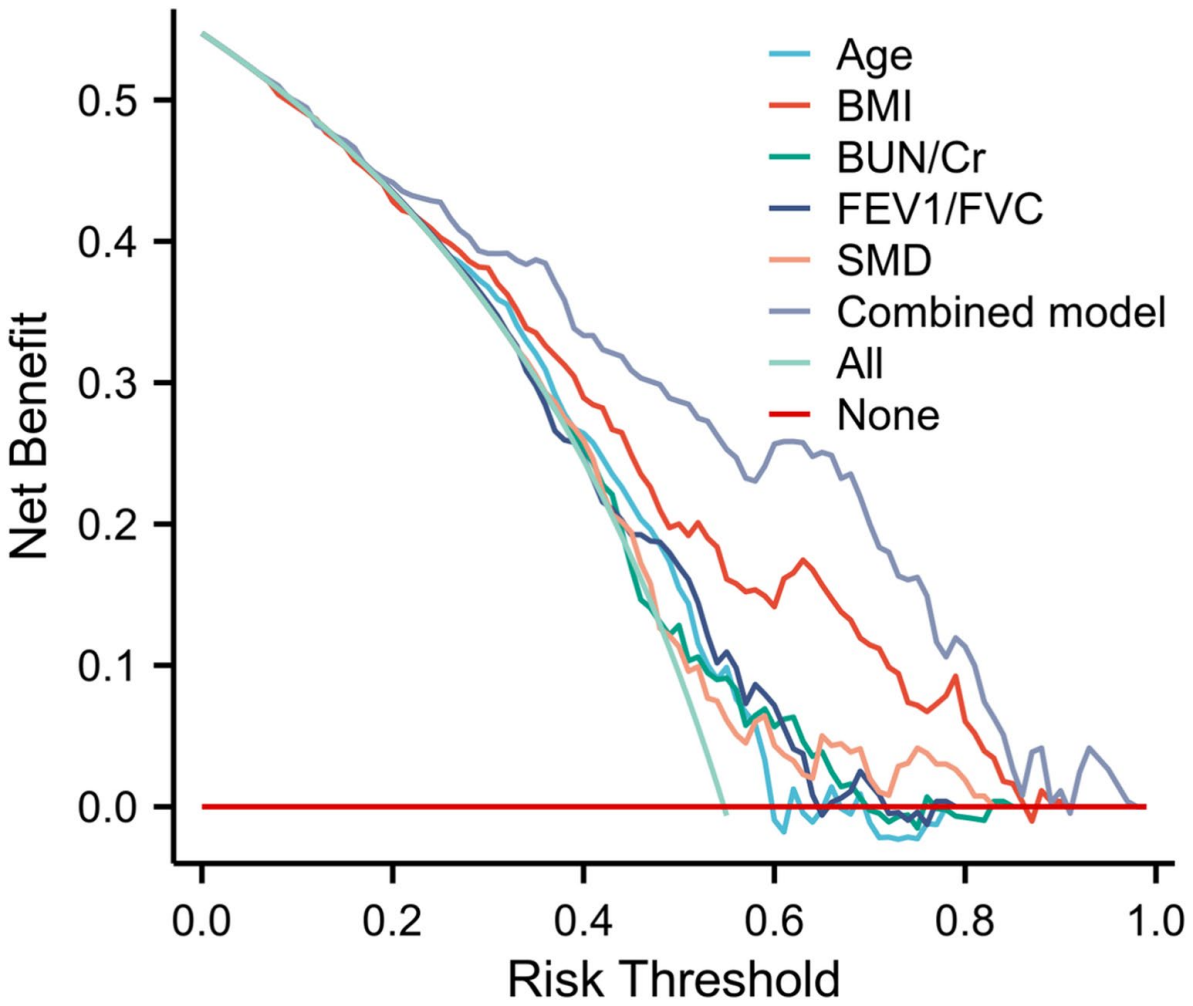
Decision curve analysis (DCA) substantiated the incremental clinical net benefit of the integrative nomogram across a broad range of threshold probabilities, outperforming individual predictive factors. BMI, body mass index; SMD, skeletal muscle density; BUN, blood urea nitrogen; Cr, serum creatinine; FEV1, forced expiratory volume in the first second; FVC, forced vital capacity.

To facilitate expedited personalized risk stratification, a graphical nomogram was engineered by integrating the five independent predictors (Figure 5). The optimal diagnostic threshold for the BUN/Cr ratio was determined to be 97.89 via Youden’s index (AUC = 0.602, Figure 6) (23). The integrative predictive framework (combined model) yielded superior discriminatory performance, characterized by an AUC of 0.823, which significantly outperformed the BUN/Cr ratio alone (*p* < 0.001). Calibration analysis showed good agreement between predicted and observed probabilities, and the Hosmer-Lemeshow test was not statistically significant (*p* > 0.05, Figure 7). Finally, DCA substantiated that the integrative model provided a greater net benefit than either the treat-all or treat-none strategies across a threshold probability range of 0.0 to 0.8 (Figure 8).

## 4 Discussion

Early identification of hospitalized COPD patients at high risk of low muscle mass is essential to reduce adverse clinical outcomes (24, 25). This study developed and internally validated a nomogram for identifying CT-defined low muscle mass using routinely available clinical variables and opportunistic chest CT-derived SMD. The final model incorporated five independent factors-age, BMI, BUN/Cr ratio, FEV1/FVC, and SMD-and demonstrated good discrimination, satisfactory calibration, and potential clinical net benefit on DCA. To our knowledge, this is among the few studies to integrate clinical, laboratory, pulmonary function, and chest CT-derived muscle quality information into a single prediction tool for estimating the likelihood of low muscle mass in this specific inpatient population.

Our findings are broadly consistent with prior evidence indicating that older age, lower BMI, poorer pulmonary function, and impaired muscle quality are associated with reduced muscle reserves in COPD. Aging is a well-established contributor to muscle loss through mechanisms including anabolic resistance, chronic inflammation, mitochondrial dysfunction, and reduced physical activity. Lower BMI likely reflects overall nutritional and metabolic vulnerability, whereas reduced FEV1/FVC may indicate more severe airflow limitation and greater systemic disease burden. In addition, lower SMD reflects poorer muscle quality and greater intramuscular fat infiltration, which may accompany or precede measurable declines in muscle quantity. Together, these findings support the biological plausibility of the final model.

The association between an elevated BUN/Cr ratio and low muscle mass is also clinically relevant. A higher BUN/Cr ratio may reflect catabolic stress, dehydration, poor nutritional status, or reduced creatinine generation related to lower muscle mass. Although the discriminatory ability of the BUN/Cr ratio alone was modest, its inclusion in the multivariable model suggests that it may provide complementary metabolic information when interpreted alongside anthropometric, pulmonary, and imaging-derived indicators. This supports the value of integrating routinely available laboratory markers into pragmatic risk assessment tools for hospitalized patients with COPD.

Recent studies also support the clinical value of imaging-based muscle assessment in acute respiratory illness. For example, Lee et al. (26) demonstrated that paraspinal muscle index predicted adverse outcomes in elderly patients with community-acquired pneumonia. Similarly, Şirin et al. (27) showed that rectus femoris and vastus intermedius muscle thickness were associated with mortality in older patients with pneumonia. These studies support the broader validity of opportunistic muscle imaging in hospitalized respiratory patients. Our study extends this concept by constructing a dedicated prediction tool that combines opportunistic chest CT-derived muscle quality with routine clinical variables to estimate the probability of low muscle mass in patients hospitalized with COPD.

From a clinical perspective, this nomogram serves as an early screening tool for hospitalized COPD patients undergoing routine chest CT. In settings where formal body composition assessment or comprehensive geriatric evaluation is not routinely available, the model may help clinicians identify patients at high risk of low muscle mass, prioritize referral for nutritional assessment, physical therapy, or pulmonary rehabilitation, and improve risk stratification and personalized care without additional cost or radiation. Importantly, the model is not intended to replace formal sarcopenia diagnosis but to support efficient triage in resource-limited inpatient settings.

Several limitations should be considered when interpreting our findings. First, the present study evaluated CT-defined low muscle mass rather than formal consensus-defined sarcopenia (28). Sarcopenia requires low muscle mass plus low muscle strength and/or physical performance, which were not available in this retrospective cohort. All findings must be interpreted within this framework. Second, low muscle mass was defined using sex-specific mean T12 SMI values derived from our own cohort, because no universally accepted T12-specific cutoff is currently available (29). These thresholds should therefore be regarded as exploratory and cohort-specific rather than externally generalizable diagnostic standards. Third, both the outcome variable (T12 SMI) and one predictor (T12 SMD) were derived from the same CT slice. Although SMI and SMD reflect different dimensions of muscle composition, namely quantity and quality, this shared anatomical source may have introduced methodological correlation and may have inflated model performance to some extent. Fourth, this was a retrospective single-center study, which may have introduced selection bias and residual confounding from unmeasured factors such as frailty, nutritional status, medication exposure, pre-admission functional capacity, and disease severity. Finally, only internal validation was performed. Therefore, the transportability and clinical utility of the model in other institutions, populations, and imaging settings remain uncertain and require external validation before broader application.

## 5 Conclusion

In conclusion, we developed and internally validated a nomogram for estimating the probability of CT-defined low muscle mass in hospitalized patients with COPD using routinely available clinical variables and opportunistic chest CT-derived SMD. The model showed promising discrimination and calibration and may assist early risk stratification in routine inpatient practice. However, because the study addressed low muscle mass rather than formal consensus-defined sarcopenia, used cohort-derived T12 SMI thresholds, and lacked external validation, the findings should be interpreted cautiously. Prospective multicenter studies incorporating muscle strength and physical performance are needed before broader clinical application.

## Data Availability

The original contributions presented in the study are included in the article/supplementary material, further inquiries can be directed to the corresponding author.

## Glossary

Alb: serum albumin
AUC: area under the receiver operating characteristic curve
BMI: body mass index
BUN: blood urea nitrogen
CI: confidence interval
COPD: chronic obstructive pulmonary disease
Cr: serum creatinine
CRP: C-reactive protein
CSA: cross-sectional area
DCA: decision curve analysis
FEV1: forced expiratory volume in the first second
FEV1% pred: percentage of predicted FEV1
FVC: forced vital capacity
GOLD: Global Initiative for Chronic Obstructive Lung Disease
Hgb: hemoglobin
LASSO: least absolute shrinkage and selection operator
NLR: neutrophil to lymphocyte ratio
OR: odds ratio
PaCO2: arterial partial pressure of carbon dioxide
PaO2: arterial partial pressure of oxygen
ROC: receiver operating characteristic
SaO2%: arterial oxygen saturation
SMA: skeletal muscle area
SMD: skeletal muscle density
SMI: skeletal muscle index
SMM: skeletal muscle mass
WBC: white blood cells.
